# Dataset concerning the hourly conversion factors for the cumulative energy demand and its non-renewable part, and hourly GHG emission factors of the Swiss mix during a one-year period (2016 and 2017)

**DOI:** 10.1016/j.dib.2020.105509

**Published:** 2020-04-15

**Authors:** Didier Vuarnoz, Sergi Aguacil Moreno

**Affiliations:** Building2050 research group, Ecole Polytechnique Fédérale de Lausanne (EPFL), Passage du Cardinal 13B, CH-1700 Fribourg, Switzerland

**Keywords:** Primary energy, GHG emissions, Life-cycle analysis, Electricity mix, Switzerland

## Abstract

The provided data are the hourly CO2-eq emission factors, and the hourly conversion factors for the cumulative energy demand and its non-renewable part for the Swiss electricity mix over one year (2016 and 2017). These data have been assessed on the base of an inventory of the technology used for electricity generation and an attributional life-cycle approach according to the methodology presented in Vuarnoz and Jusselme (2018). Compared with Vuarnoz and Jusselme [2], electricity imports from Italy to Switzerland are not neglected anymore, and lead to more accurate output data. The utility of the proposed data lies in the multiple possible applications. The presented data are necessary for conducting a life cycle assessment of all processes and products using electricity in Switzerland. Moreover, the presented data could serve as a sustainable benchmark of electricity when implementing renewable energy systems and energy storage [7]. Because of their temporal accuracy, the hourly conversion factors enable the development of energy management strategies taking into account the time-dependent life cycle impacts. Finally, they can be used for the quantitative follow-up of the decarbonization process of the grid electricity at the national level over a given lapse of time.

Specifications table

**Table:** 

Subject	Energy
Specific subject area	Life cycle assessment of electricity, i.e. hourly emission factor, hourly conversion factors for the cumulative energy demand and its non-renewable part
Type of data	Excel file
How data were acquired	Application of the Input-Output assessment model described in [1] for the analyzed period (01/01/2016–31/12/2016 and 01/01/2017–31/12/2017) with hourly input data from: -for the inventory of technology involved in electricity generation: Entsoe Transparency Platform [3] for Switzerland, Germany, Austria, Italy and France -for the amount of electricity imports: Swissgrid [4] for Switzerland Eurostat. (2018) [5] -for the technology-specific conversion factors: KBOB database [6]
Data format	Raw
Parameters for data collection	The life cycle assessment is performed with “cradle-to-grave” system boundaries. Transport and distribution losses are included in the assessment.
Description of data collection	The reference time is GMT+1. The data are given for a functional unit of 1 kWh of electricity
Data source location	Switzerland
Data accessibility	https://data.mendeley.com/datasets/m5cd9spsrk/2 http://dx.doi.org/10.17632/m5cd9spsrk.2
Related research article	The methodology used to assess the dataset is described in: D.Vuarnoz, T.Jusselme, Temporal variations in the primary energy use and greenhouse gas emissions of the electricity provided to the Swiss grid. Energy 161: 573–582 [1], https://doi.org/10.1016/j.energy.2018.07.087

## Value of the data

• The dataset can be directly used to compute Life Cycle Assessment (LCA) of processes and products using electricity.
• The dataset can serve to develop time-dependent strategies of electricity use for primary energy optimization and greenhouse gases emission mitigation.
• The dataset can be compared with the dataset of electricity mixes from different regions/countries.
• The dataset can serve as a benchmark, e.g. for the same national grid mix during other period of time, and for a sustainable implementation of renewable energy system and energy storages.

## 1 Data description

The data provided within this article consist of hourly conversion factors for the cumulative energy demand (CED) and its non-renewable part (CEDnr), both in [MJ_oil-eq_/kWh], as well as the CO_2-eq_ emission factors (GWP) in [kg CO_2-eq_/kWh] of the electricity provided by the Swiss mix during a one-year period (01/01/2016–31/12/2016 and 01/01/2017–31/12/2017). See the .xlsx file.

## 2 Experimental design, materials, and methods

The methodology used to generate the dataset presented in this article is detailed in [1]. The method considers an input-output model. Any pre-treatment of the input data has been performed and no filter has been applied to the obtained dataset. Original input data used for the assessment originates from different sources and consist of hourly inventories of domestic productions, hourly electricity imports/exports and technology-specific conversion factors. For each domestic production, data from the inventory are (1) the energy generation per hour (kWh/h) and (2) the types of technology used. Regarding the domestic productions, the inventory of the technologies involved during each hour has been provided by [3]. The technology-specific conversion factors used for the assessment are those from the KBOB database [6]. With regard to the electricity imports, hourly values of the Swiss imports have been provided by [4]. French, Austrian, Italian and German imports have been assumed to be constant over one year, and corresponding to the mean annual values given in [5].

## Declaration of Competing Interests

The work presented in this paper has been funded by the State of Fribourg (message du Conseil d'Etat au Grand Conseil 2014-DEE-22) and EPFL.

## Appendix Supplementary material

Image, application 1
